# Response of pheochromocytoma neuronal cells to varying intensity of continuous wave terahertz radiation

**DOI:** 10.1107/S1600577525008227

**Published:** 2025-10-16

**Authors:** Denver P. Linklater, Palalle G. Tharushi Perera, Zoltan Vilagosh, Alexis Perez-Gonzalez, Phuc H. Le, Tanavi Sharma, Michael G. Leeming, Nicholas A. Williamson, Dominique Appadoo, Rodney Croft, Elena P. Ivanova

**Affiliations:** a Graeme Clark Institute of Biomedical Engineering, Faculty of Engineering and Information Technology The University of Melbourne Parkville VIC3010 Australia; b School of Science RMIT University PO Box 2476 Melbourne VIC3001 Australia; c Melbourne Cytometry Platform The University of Melbourne Parkville VIC3010 Australia; d Department of Microbiology and Immunology The University of Melbourne, at The Peter Doherty Institute of Infection and Immunity Parkville VIC Australia; e Melbourne Mass Spectrometry and Proteomics Facility, Bio 21 Molecular Science and Biotechnology Institute The University of Melbourne Parkville VIC3010 Australia; f THz/Far-Infrared Beamline Australian Synchrotron Clayton VIC3168 Australia; g School of Psychology, Australian Centre for Electromagnetic Bioeffects Research University of Wollongong Wollongong NSW2522 Australia; SESAME, Jordan

**Keywords:** terahertz synchrotron radiation, electromagnetic energy, cell permeability, proteomics

## Abstract

The effects of varying intensities of Australian Synchrotron source terahertz (THz) radiation on pheochromocytoma (PC 12) neuronal cells were investigated. PC 12 cells were exposed to THz radiation at beam incident power intensities of 0.25 W m^−2^ (low intensity, LI), 0.5 W m^−2^ (medium intensity, MI) and 1 W m^−2^ (high intensity, HI) for 10 min. After exposure, the morphological and physiological status of the cells was evaluated using scanning electron microscopy (SEM) and confocal laser scanning microscopy. SEM imaging revealed that, after exposure to LI THz radiation, the cells exhibited membrane protrusions (blebs) measuring 70–120 nm in diameter. In contrast, cells exposed to HI THz radiation demonstrated increased uptake of FITC–dextran and nanospheres. Analysis of single-cell populations counterstained with 4′,6-di­amidino-2-phenyl­indole (DAPI) showed a decrease in the proportion of DAPI-positive cells, with approximately 90, 80 and 50% remaining positive after exposure to LI, MI and HI THz radiation, respectively. However, only a slight increase in the proportion of dead cells was observed at varying THz intensities. Proteomic analysis of the cell changes following exposure to LI and HI THz irradiation indicated that THz radiation activated the CaN complex and upregulated genes involved in ribosome biogenesis and DNA damage repair.

## Abstract 2

PC 12 neuron-like cells were exposed to terahertz (THz) radiation of variable beam incident power intensity from 0.25 to 1.0 W m^−2^. THz irradiation activated the CaN complex, ribosome biogenesis and DNA damage reparation.

## 1. Introduction

Terahertz (THz) radiation occupies a space in the electromagnetic spectrum between millimetre waves and infrared light, with a frequency of 0.1–10 THz and wavelength of 0.03–3 mm (Soghomonyan *et al.*, 2016 ▸). THz waves are non-ionizing due to their low photon energy (Weightman, 2012 ▸; Vilagosh *et al.*, 2022 ▸). THz photon energy is comparable to the rotational and vibrational energy levels of many organic and biological macromolecules. This property makes THz waves useful for detecting specific cell types and macromolecules by characterizing their unique absorption spectra. Furthermore, THz wavelengths can penetrate a wide variety of materials. Their low photon energy makes them ideal for the non-destructive, real-time detection of biological materials, as they do not cause tissue ionization (Zhang *et al.*, 2021 ▸); for example, in the early detection of cancers (Shi *et al.*, 2020 ▸). While it is effective for use in medical diagnostics and theranostics, there is currently insufficient data to support its safety. The lack of radiation safety studies means that the current THz radiation safety limits rely on extrapolating the safety data obtained for the neighbouring millimetre and infrared wavelengths. Furthermore, the bioeffects of THz radiation may depend on the THz source, and whether it is a continuous wave or pulsed exposure (Cherkasova *et al.*, 2021 ▸). Current THz emitters that may have practical use in industry allow the generation of incident power from nanowatts to kilowatts, have tunable bandwidth and emit THz frequencies anywhere from 1 to 10 THz (Cherkasova *et al.*, 2021 ▸).

Documented cell response to THz waves is proposed to be due to two distinct mechanisms of action: heating due to the strong THz absorption by water and nonthermal effects (Alexandrov *et al.*, 2010 ▸; Cheon *et al.*, 2019 ▸; Chitanvis, 2006 ▸). Both theoretical and experimental data reported in the literature suggest that the THz effects can cause changes in DNA molecules and gene expression due to local breaks in the hydrogen bonds of DNA chains (Alexandrov *et al.*, 2010 ▸; Cheon *et al.*, 2019 ▸; Chitanvis, 2006 ▸). Usually, these resonant interactions have been observed using high-power THz sources.

Our ongoing investigations of the biological effects of high-frequency electromagnetic fields have determined that exposure of several prokaryotic and eukaryotic cell types to 18 GHz induces reversible cell membrane permeation (Perera *et al.*, 2022 ▸; Perera *et al.*, 2018 ▸; Perera *et al.*, 2021 ▸) in taxonomically diverse bacteria and eukaryotic cell lines (Perera *et al.*, 2024 ▸; Shamis *et al.*, 2012 ▸; Nguyen *et al.*, 2015 ▸; Nguyen *et al.*, 2016 ▸; Perera *et al.*, 2018 ▸). In addition to reversible membrane permeation, genetic transformation was also reported using electromagnetic energy (EME) of 18 GHz in a competent bacterial strain with no detrimental effects on cell viability (Perera *et al.*, 2024 ▸). Previous assessments of PC 12 exposures to 10 min synchrotron source (SS) THz beam exhibited reversible permeability with no significant changes to cellular metabolism (Perera, Appadoo *et al.*, 2019 ▸; Perera, Bazaka *et al.*, 2019 ▸). PC 12 neuronal differentiation remained unaffected among the exposed groups. However, more information is required to ascertain the physiological response of PC 12 cells to increasing intensity of THz exposure. Therefore, in this work, we aimed to study the physiological and proteome changes in PC 12 cells exposed to varying intensities of THz radiation.

## 2. Material and methods

### 2.1. PC 12 cells growth conditions

Pheochromocytoma cells (PC 12) were purchased from the American Type Culture Collection (ATCC, USA). PC 12 cells were cultured in complete Gibco RPMI medium (Thermo Fisher Scientific, Australia) supplemented with 10% Gibco horse serum (Thermo Fisher Scientific, Australia, HS), 5% Gibco foetal bovine serum (Thermo Fisher Scientific, Australia, FBS) and 1% Gibco penicillin/streptomycin (Thermo Fisher Scientific, Australia, PS). The cells were cultured at 37°C in a 95% humidified incubator with 5% CO_2_. The medium was changed every two days and passaged accordingly when the confluence reached 90%.

### 2.2. Sample preparation and SS THz exposure

The PC 12 cells were exposed to broadband SS EME-THz using the Far-IR/THz beamline at the Australian Synchrotron (Clayton, Victoria, Australia). The cells were placed at the beam extraction port (BEP) to maximize exposure. A monolayer of PC 12 cells was achieved using 30 µl (6.0 × 105 cells) in phosphate-buffered saline (PBS) on a polyethyl­ene (PE) film using an O-ring sealed with grease. The SS EME-THz beam intensity at the sample was 1.0 W m^−2^, with a frequency dispersion outlined in Fig. S1 of the supporting information. The beam was centred on 4.0 THz with half maxima at 2.0 and 8.0 THz. The intensity fell rapidly under 0.5 THz, but a long, low-intensity infrared tail extended beyond 430 THz.

The beam intensity was adjusted using the diverting mirror at the extraction port in different positions. This allowed the beam to either fully irradiate the sample or be diverted to the resident Si Bolometer. Three settings were used: the ‘high intensity’ (HI) setting had the mirror position 9.5 (maximum beam irradiation) with an Si Bolometer intensity count = 500, which is the Si Bolometer background with no beam. The ‘medium intensity’ (MI) setting used the mirror position 6.5, giving an Si Bolometer intensity count of 11400, representing 50% beam intensity. The ‘low intensity’ (LI) used mirror position 5.5, Si Bolometer count = 17000, which results in 25% beam exposure on the sample, since 75% of the beam was now going to the Si Bolometer. The settings are outlined in Table 1 ▸.

**Figure 1:**
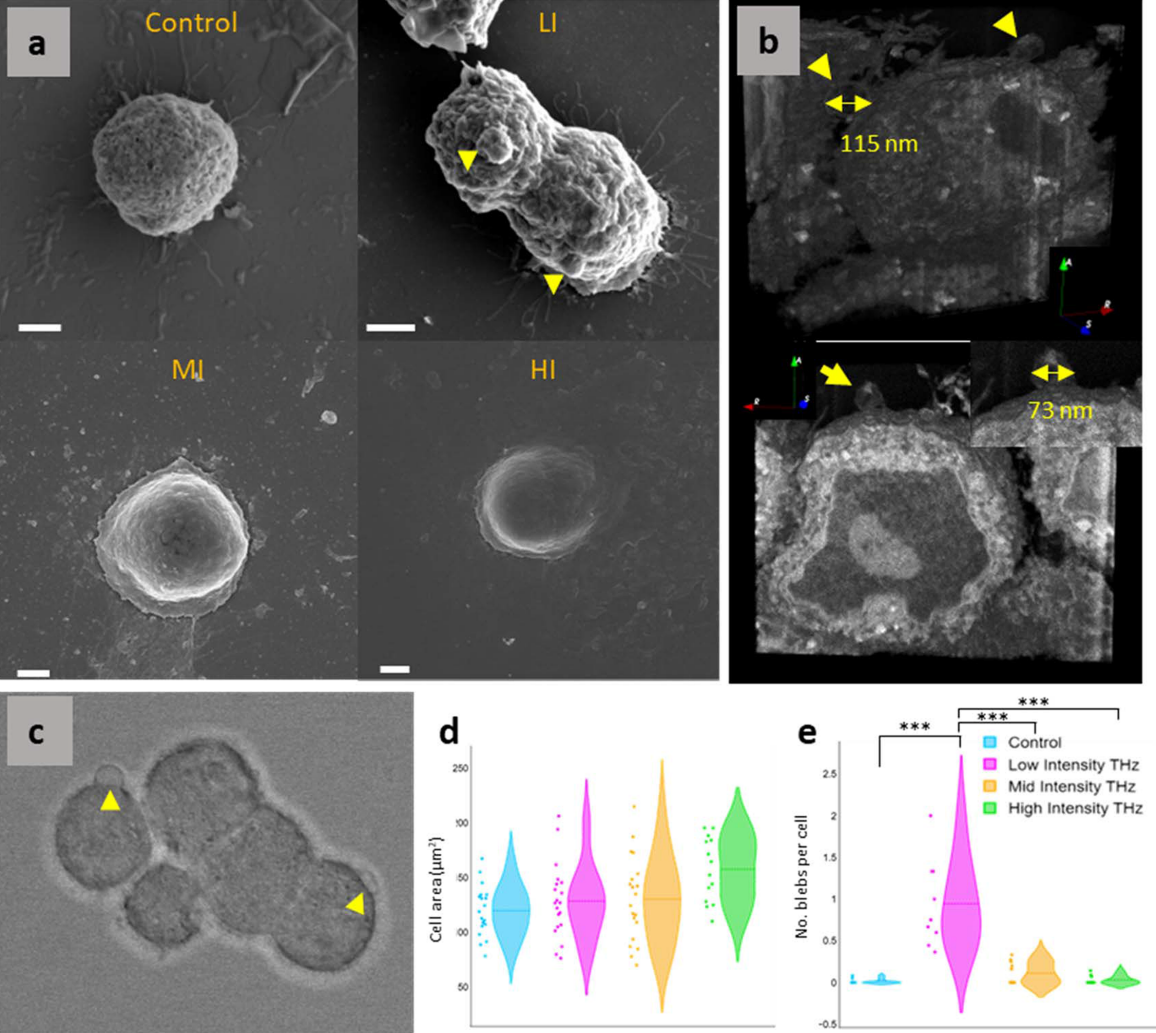
Morphology of PC 12 cells exposed to THz. (*a*) Representative SEM micrographs of control PC 12 cells and PC 12 cells exposed to LI, MI and HI THz for 10 min. Cells exposed to LI THz are observed to exhibit significant membrane blebbing. (*b*) 3D volume reconstruction of serial blockface FIB-SEM of PC 12 cells with surface blebs exposed to LI THz. Yellow arrows indicate blebs. (*c*) Phase contrast images of PC 12 cells with blebs. (*d*) Graph of the cell area distribution after different exposure intensities, as determined by CLSM imaging. (*e*) Size analysis of cell blebs for each exposure group. Statistical significance is denoted by *** *p* < 0.01.

The temperature of the cells was recorded before, during and after exposure for all treatment groups [Figs. 1 ▸(*a*)–1 ▸(*f*)]. The temperature was recorded every minute for 10 min. After the exposure, the sample was collected from the PE film and analysed. Three repeats were carried out for post-exposure analysis. Furthermore, few independent technical replicates were performed over time. An IR camera (SNOOU, Multifunction Infrared Thermal Imaging) was used to capture heat maps of the sample at *T* = 0 and *T* = 10, along with the temperature detected at the point of focus. The IR camera had a screen resolution of 320 × 240 pixels, the temperature measurement range −40°C to 300°C and a measurement accuracy of ±2°C. Approximately 5−10 images were taken at LI, MI and HI.

### 2.3. Cellular uptake of silica nanospheres

FITC–silica nanospheres (NS) with a diameter of 23.5 ± 0.2 nm (FITC) (Corpuscular, Cold Spring, NY, USA) were used to investigate the permeability of the PC 12 cells immediately after THz radiation exposure. The NS were added at a concentration of 100 µg ml^−1^. After a 10 min incubation, samples were washed twice using PBS and centrifugation at 1300 rpm for 5 min at 25°C. The procedure was repeated for untreated controls. Cell uptake was visualized by confocal laser scanning microscopy (CLSM) using a Fluoview FV10i-W inverted microscope (Olympus, Tokyo, Japan).

### 2.4. Cellular uptake of fluorescent dextran probes

Fluorescent FITC–Dextran Probes (Merck, Melbourne, Australia) were added to SS THz exposed samples and relevant controls at a final concentration of 1 mg ml^−1^ in PBS and incubated for 10 min. Afterwards, samples were washed twice using PBS and centrifugation at 1300 rpm for 5 min at 25°C. Cells were counter-stained with 4′,6-di­amidino-2-phenyl­indole (DAPI) 5 µ*M*. FITC–dextran uptake was confirmed using CLSM and flow cytometry (FC) (detailed below).

### 2.5. Flow cytometry

FC was performed using a Cytek Aurora Flow Cytometer. FC calibration was conducted using a reference bead mix. The FC tubing was washed in 10% bleach to remove any particles that might have remained within the tubing. Bleach and PBS washes were conducted before each sample change. The measurements were adjusted to a flow rate of 25 µl min^−1^, stop condition: 5000 events. The voltage was adjusted accordingly and the fcs files were generated using the *Invitrogen Attune Nxt Flow Cytometer* (Thermo Fisher Scientific). The resulting fcs files were processed with the *Floreada* analysis tool available at https://floreada.io (accessed on 11 November 2022). To exclude debris from the analyses, which displayed low FSC values, gating in the FSC versus SSC plot was performed for every determination.

### 2.6. Volume block-face imaging analysis of nanosphere internalization

After 10 min of SS THz exposure, cell suspensions were pelleted by centrifugation at 1300 rpm for 5 min at 25°C. The cell pellet was conditioned with 0.1 *M* sodium cacodylate buffer (pH 7.4). The cell pellet was then resuspended in 4% paraformaldehyde and 2.5% glutaraldehyde overnight at 4°C and washed three times in cacodylate buffer for 10 min each. The cells were post-fixed in 1% osmium tetroxide (OSO_4_) and 1.5% potassium ferrocyanide for 1 h followed by washing three times in distilled water for 10 min each. The cells were dehydrated using a graded series of ice-cold ethanol (50, 70 and 90%) for 15 min each. The cells were further dehydrated by passing through 100% ethanol twice followed by 100% acetone twice for 30 min each. The cells were then infiltrated with a 1:1 ratio of acetone:Spurr’s resin (Spurr, 1969 ▸) mixture overnight. After that, the cells were completely exchanged in 100% Spurr’s resin twice for 3 h each time, under vacuum. The resin samples were polymerized at 70°C for 48 h. The final block was trimmed and serial block face imaging was performed using a SCIOS FIB-SEM equipped with a gallium ion source.

### 2.7. Cell morphology

PC 12 cell morphology was examined using a scanning electron microscope FeSEM SUPRA 40VP (Carl Zeiss, Jena, Germany) at an accelerating voltage of 3 kV. A 100 µl aliquot of cells in PBS was placed on poly l-lysine-coated glass coverslips (ProSciTech, Kirwan, Australia). The PC 12 cells were fixed in 2.0% paraformaldehyde and 2.5% glutaraldehyde for 30 min. The cells were then dehydrated using a graded ethanol series (20, 40, 60, 80 and 100%) for 15 min each. Before imaging, the fixed cells were gold coated (7 nm thick) using a NeoCoater MP-19020NCTR (Jeol, Tokyo, Japan).

### 2.8. Cell viability

The viability of PC 12 cells was determined using the LIVE/DEAD Viability/Cytotoxicity Kit (Invitrogen) according to the manufacturer’s protocol. CLSM was used to image the number of viable cells; approximately ten fields of view were analysed per sample type.

### 2.9. Proteomics analysis

#### 2.9.1. Protein extraction

Cell pellets were suspended in 5% *w*/*v* sodium do­decyl sulfate (SDS) (Merck, Australia) in 100 m*M* tri­ethyl­ammonium bicarbonate (TEAB) (Merck, Australia), vortexed and sonicated for 20 min. Afterwards, the suspension was centrifuged at 13000*g* for 8 min to pellet any remaining insoluble components. Then, the supernatant was transferred into a new Eppendorf tube. BCA assay (PierceTM BCA Protein Assay kit, ThermoFisher Scientific, USA) was performed to estimate the protein concentration as at least 50 µg of proteins from each sample was taken for further extraction process.

Reduction and alkyl­ation were performed in 2 ml microcentrifuge tubes using 10 m*M* and tris­(2-carb­oxy­ethyl) phosphine (TCEP) (Merck, Australia) at 55°C for 15 min and 50 m*M* iodo­acetamide (IAA) at room temperature under dark conditions for 45 min. The SDS solution was then acidified with 1.2% *v*/*v* phospho­ric acid (Merck, Australia). The acidified solution was mixed with S-trap binding buffer (100 m*M* TEAB in 90% *v*/*v* HPLC grade MeOH) to create a colloidal protein suspension. The samples were loaded onto the S-trap mini-columns and centrifuged at 4000*g* for 30 s, trapping the proteins in the S-trap column matrix. The trapped proteins were washed four times with 150 µl of S-trap binding buffer with centrifugation at 4000*g* for 30 s between each wash. Protein digestion was performed by adding 20 µl of PierceTM Trypsin Protease (ThermoFisher Scientific, USA) (50 m*M* TEAB containing a 1:50 *w*/*w* ratio of trypsin to protein) and incubating overnight at 37°C.

Following the incubation period, peptides were separated using a sequence of elution solutions, which consisted of TEAB (50 m*M*), 0.2% formic acid and 50% aceto­nitrile (ACN) (Merck, Australia). These eluted peptides were combined and centrifuged using the SpeedVac concentrator (ThermoFisher Scientific, USA). This step eliminated any remaining ACN before the peptides underwent freeze-drying. Once dried, the peptides were reconstituted in a solution containing 2% ACN and 0.05% tri­fluoro­acetic acid (Merck, Australia) and subsequently loaded into the liquid chromatography-tandem mass spectrometry (LC-MS/MS) instrument for analysis.

#### 2.9.2. Data analysis

Data-independent acquisition (DIA) was carried out on the Orbitrap Eclipse mass spectrometer (Thermo Fisher Scientific, San Jose, CA, USA). The settings of nano electrospray voltages, ion funnel RF and capillary temperature were 1.9 kV, 30% and 275°C, respectively. A survey scan with the *m*/*z* range 350–1400, a resolution of 120000, an automatic gain control (AGC) target of 1×10^6^ and a maximum ion trapping time of 50 ms was performed before 50 DIA windows with an *m*/*z* isolation window of 13.7, precursor ion *m*/*z* range 361–1033, MS/MS scan range *m*/*z* 200–2000, a resolution of 30000, an AGC of 1×10^6^, a maximum ion trapping time of 55 ms and normalized collision energy of 30%. Data were processed using *Spectronaut 16.0* (Biognosys, Zurich, Switzerland) with a direct DIA pipeline and the default BGS Factory settings. Trypsin as the enzyme, acetyl­ation at the protein N-terminal and oxidation at me­thio­nine as variable modification, carbamido­methyl at cysteine as fixed modification, 1% false discovery rate at PSM, peptide and protein levels were selected. The proteome databases from UniProt were downloaded for comparative analysis using *MaxQuant* (v2.4.8.0; developed by Max Planck Institute of Biochemistry, Germany). *Perseus* (v1.6.15.0; developed by Max Planck Institute of Biochemistry, Germany) was used for un­supervised hierarchical clustering analysis and two sample *t*-tests [*p* < 0.05 as well as log2(fold change) ≥ 1 or ≤ −1 were required for significance]. The protein functional annotations including biological processes and molecular functions were analysed using *GeneCodis4*.

## 3. Results

### 3.1. Experimental setup of the PC 12 cells exposed to varying intensities of SS THz radiation

The frequency of the SS THz beam at the Australian Synchrotron ranges from 0.5 THz to >20.0 THz with a variable intensity. The spectrum of the intensity beam is centred on 4.0 THz with the half-width maxima occurring at 2.0 THz and 8.0 THz (Fig. S1 of the supporting information). The current configuration of the SS THz beam delivers an approximate power of 0.058 mW over a 4.5 mm radius spot size, giving a total intensity of ∼1.25 W m^−2^ (0.125 mW cm^−2^) at the BEP. After adjustments for the losses due to the sample holder, the intensity reduces to 1.0 W m^−2^.

Neuronal cells have an extraordinary property of maintaining the characteristic features of their tissues of origin (Oprea *et al.*, 2022 ▸; Perera, Bazaka *et al.*, 2019 ▸; Wiatrak *et al.*, 2020 ▸), enabling them to be a physiologically and biologically relevant model for *in vitro* studies (Orlowska *et al.*, 2017 ▸; Wiatrak *et al.*, 2020 ▸). PC 12 cells possess the ability to proliferate and differentiate in response to nerve growth factor while expressing neuronal biomarkers and forming axons and dendrites (Wiatrak *et al.*, 2020 ▸). PC 12 neuron-like cells were exposed to THz radiation at LI, MI and HI for periods of 10 min. The beam intensity was adjusted at the BEP by manual movement of a mirror into the beam path and the intensity was digitally recorded. The HI setting with an Si bolometer intensity count of 500, which is the Si bolometer background with no beam. The MI setting gives an Si bolometer intensity count of 11400, which represents 50% beam intensity. The LI setting gave a Si bolometer count of 17000 resulting in 25% of the beam exposing the sample. These settings can confirm a beam incident power intensity of 0.25 W m^−2^ for LI, 0.5 W m^−2^ at MI and 1 W m^−2^ for HI. The exposure was conducted at the BEP as described previously (Perera *et al.*, 2023 ▸). The control cells were handled near the exposed ones but were not irradiated by THz waves. The temperature was recorded at HI where the starting temperature (*T* = 0) was recorded to be 26.9°C and *T* = 10 was 28.6°C [Figs. 1 ▸(*c*) and 1 ▸(*f*)], the temperature change was ±1.7°C among all the experimental groups hence considered to be negligible. After exposure, the retrieved cells were assessed for morphological and physiological changes using electron microscopy and CLSM.

**Figure 2:**
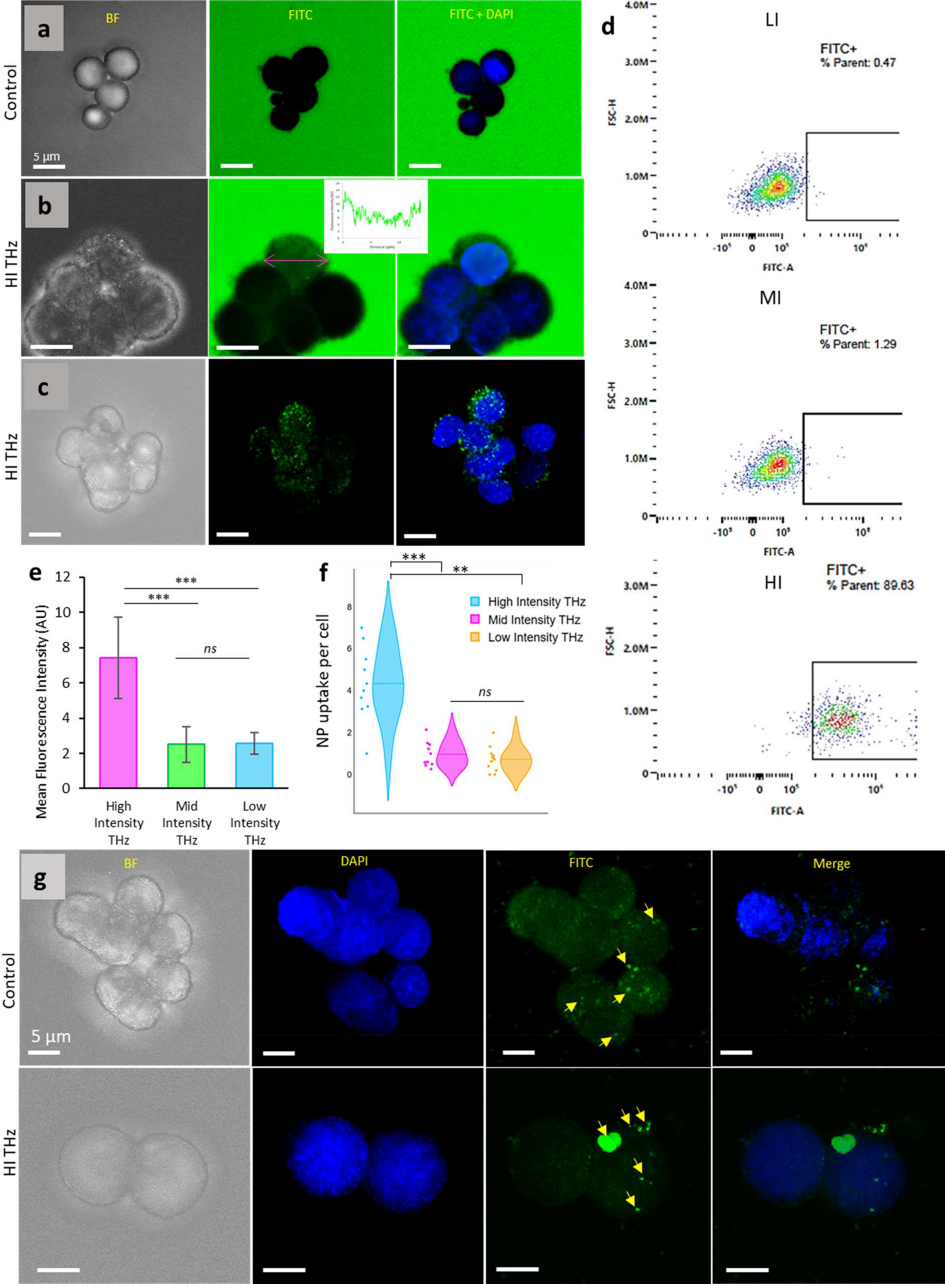
FITC–dextran uptake by PC 12 cells exposed to variable intensity THz for 10 min. (*a*)–(*c*) CLSM micrographs of (*a*) untreated cells and (*b*) cells treated with HI THz and immediately placed in fluorescein dextran. No uptake is visible in control cells whereas exposed cells show internalized green fluorescence. The inset graph shows a fluorescence intensity profile for a single cell. (*c*) CLSM micrographs of PC 12 cells treated with HI THz and immediately placed in fluorescein dextran for 10 min and then washed. (*d*) FC scatter plots showing the population of FITC-positive single live cells for cells exposed to LI, MI and HI THz for 10 min, respectively. (*e*) Mean fluorescence intensity of cells exposed to increasing intensity of THz radiation and incubated (10 min) in fluorescein dextran, normalized to the control. No difference in fluorescence intensity of the cells was observed for LI–MI THz-exposed populations. (*f*) FITC SiO_2_ NS uptake per cell for PC 12 exposed to HI, MI and LI THz radiation, normalized to the control. Statistical significance is denoted by *** *p* < 0.01, ** *p* < 0.05, ns = non-significant. (*g*) CLSM analyses of FITC–SiO_2_ NPs in non-exposed PC 12 cells (top panel) and PC 12 cells following THz exposure (bottom panel). Yellow arrows indicate NPs.

Scanning electron microscopy (SEM) imaging revealed morphological changes in cells exposed to 10 min of 0.25–1 W m^−2^ THz [Fig. 1 ▸(*a*)]. The cells exhibited a larger cell area; however, the difference was found to be statistically non-significant [Fig. 1 ▸(*h*)]. The presence of cytosolic leakage in PC 12 exposed to HI THz was also observed [Fig. 1 ▸(*g*)]. Interestingly, those cells exposed to LI (0.25 W m^−2^) THz exhibited numerous blebs on the cell membrane (Fig. 2 ▸). Volume imaging and cross-sectional analysis revealed that the blebs were between 70 and 120 nm in diameter and were membrane protrusions only (they did not contain cellular or inorganic material) [Figs. 2 ▸(*d*)–2 ▸(*f*)]. PC 12 cells exposed to LI THz possessed an average of 1.0 ± 0.5 bleb per cell, whereas cells in all other groups, including the control, exhibited an average number of blebs of ≤0.1.

### 3.2. Membrane permeability

To test the hypothesis that SS THz beam variable intensities may influence cell membrane permeabilization, immediately following irradiation, the PC 12 cells were introduced into buffer supplemented with FITC–dextran, a green, fluorescent molecule of 150 kDa with a hydro­dynamic radius of 17 nm that does not penetrate intact cells (van Duinen *et al.*, 2017 ▸), and 25 nm FITC conjugated SiO_2_ NS.

FC and CLSM were used to confirm the uptake of FITC–dextran in PC 12 cells (shown in Figs. 2 ▸ and S4). Cells exposed to 1 W m^−2^ THz possessed a significant increase (*p* < 0.01) in cell mean fluorescence intensity compared with the other treatment groups. Similarly, FC only detected FITC in HI THz-exposed PC 12 cells, evidence of the uptake of fluorescein dextran. Cells exposed to LI or MI THz did not uptake FITC–dextran (Fig. S2). Specifically, there were only 0.47% and 1.29% FITC positive cells amongst the live cell population at 0.25 and 0.5 W m^−2^, respectively. Uptake of FITC–SiO_2_ NS was determined by CLSM imaging. Interestingly, FITC–SiO_2_ NS uptake was commensurate with the intensity of THz radiation delivered to the cells, *e.g.* cell uptake when exposed to HI was an average of 4 SiO_2_ NS per cell, as determined from CLSM images, whereas those cells exposed to MI and LI THz radiation internalized only ∼1 NS per cell [Fig. 2 ▸(*f*)]. The low number of NS internalized was below the detection limit of a high-throughput technique such as FC. Therefore, we hypothesize that HI THz radiation of 10 min is sufficient to permeabilize the membrane to larger nano-objects, not only small molecules as we have shown previously (Perera, Bazaka *et al.*, 2019 ▸).

**Figure 3:**
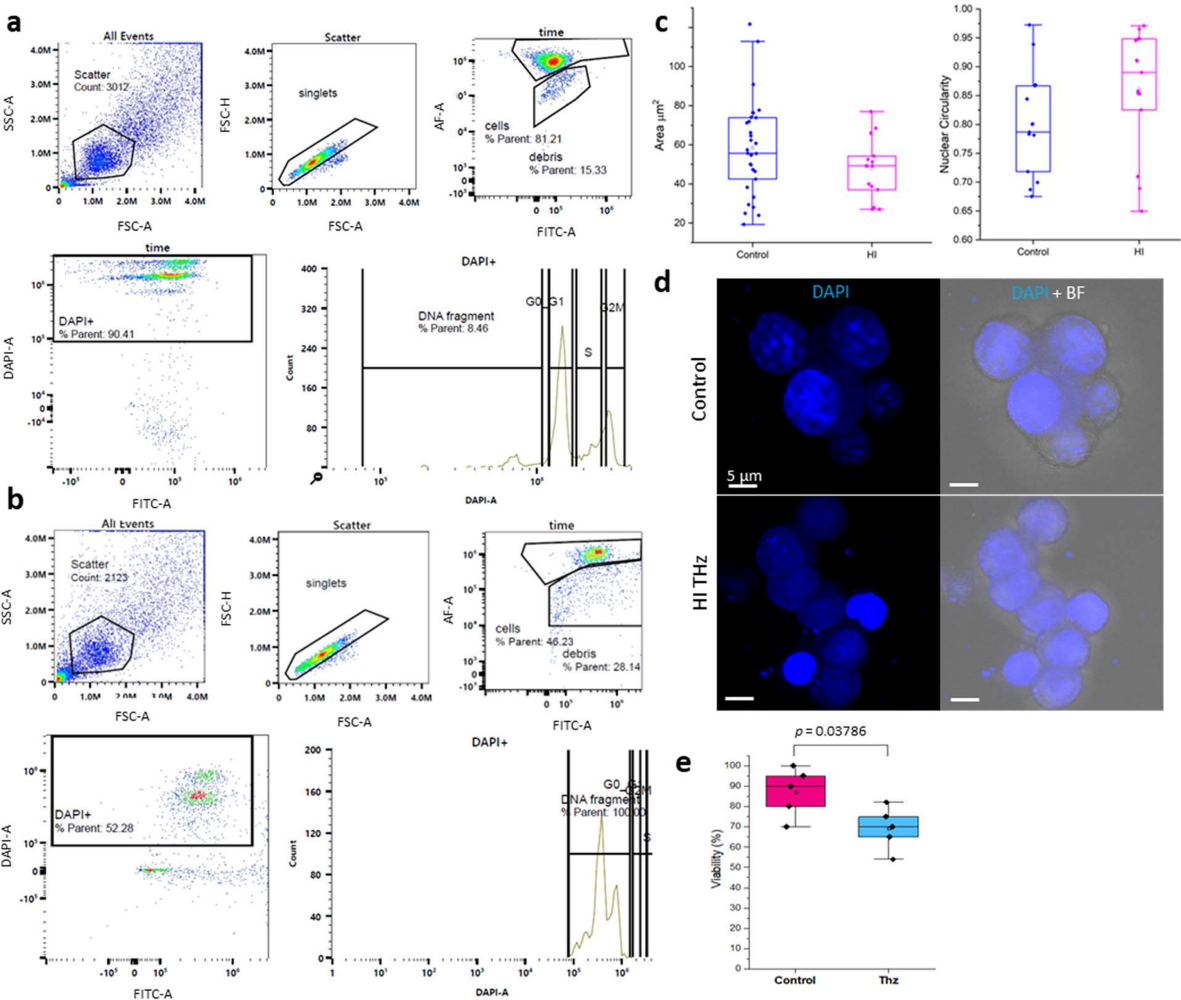
THz radiation-induced changes to DNA. FC analysis of PC 12 cells labelled with DAPI before (*a*) and after (*b*) HI THz exposure. Cells were gated using FSC versus SSC. Then single cells were gated using FSC-H versus FSC-A. Live cells were gated and cell cycle analysis was performed on DAPI-positive (+) cells. (*c*) Nuclear morphometric analysis showing quantification of nuclear area and circularity of control PC 12 and PC 12 exposed to HI THz. (*d*) Representative CLSM micrographs of control PC 12 cell nuclei (DAPI; blue) and nuclei of cells exposed to HI THz. (*d*) Viability of control PC 12 cells and cells exposed to HI THz.

The analysis of the scatter plots was performed to derive cell cycle data based on nuclear staining. As shown in Fig. 3 ▸(*a*), when the single, live cell population was considered, there was a decreasing proportion of DAPI-positive cells in response to HI THz irradiation. Changes in DNA were resolved in approximately 8% of control cells compared with 100% of cells exposed to HI THz amongst the DAPI-positive cell population. Fluorescent imaging of cells irradiated with 1 W m^−2^ showed a reduced nucleus area [Fig. 3 ▸(*c*)] compared with the non-irradiated control cells. Nevertheless, an assessment of cell viability post-HI THz exposure revealed that 70% of the cells exposed to HI THz were viable. Indeed, analysis of the singlet cell population isolated by FC showed no increase in the proportion of dead cells, or debris, under varying levels of THz intensity exposure. Approximately 30% of isolated (singlet) cells were considered dead or debris (Fig. S5). These data are corroborated by analysis of cell viability by live/dead staining that showed that 70% of cells remained viable following 10 min of HI THz exposure [Fig. 3 ▸(*e*)].

### 3.3. Proteome changes in PC 12 cells in response to variable intensity SS THz radiation

**Figure 4:**
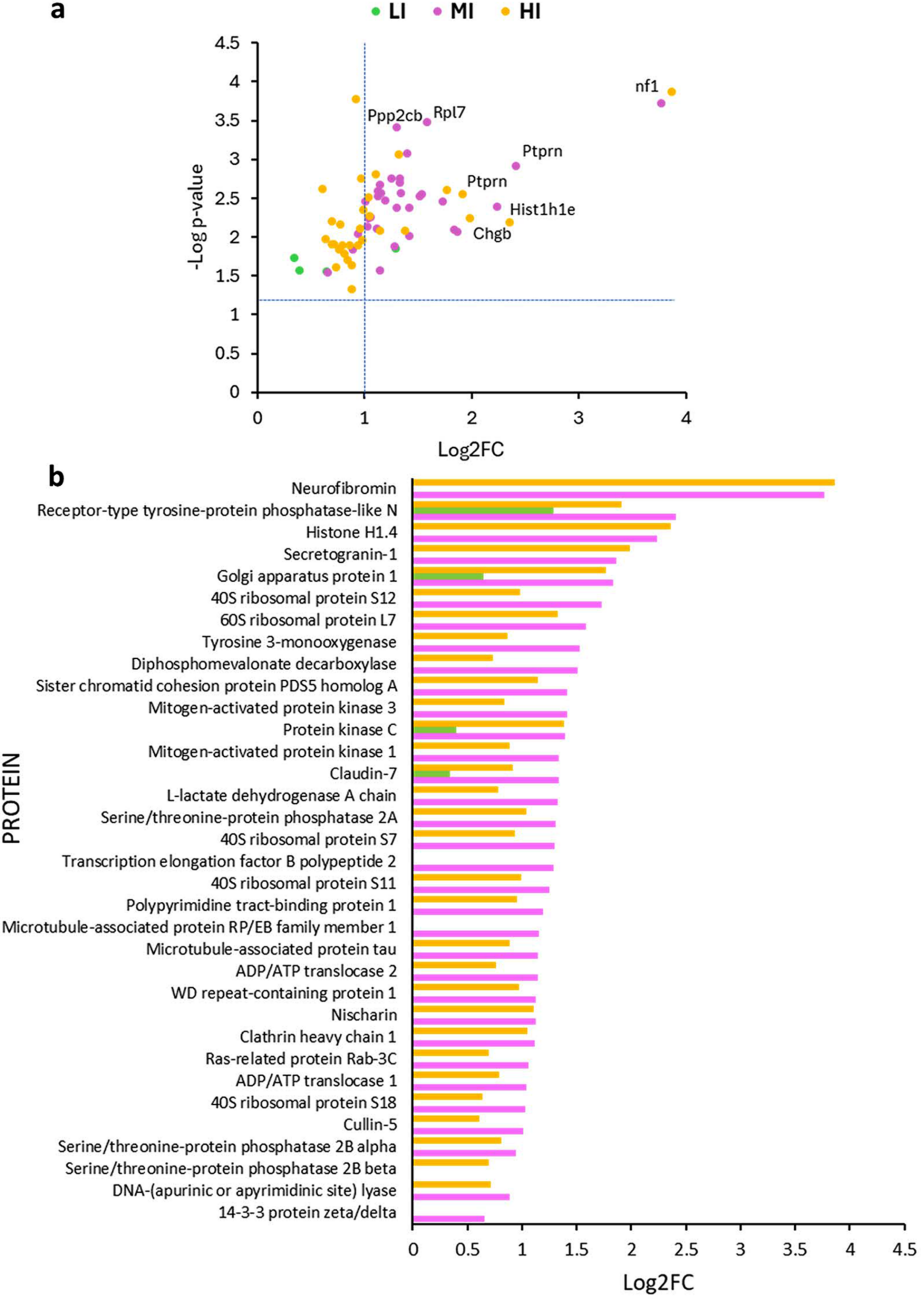
Proteomics analysis of PC 12 cells exposed to LI–HI THz. (*a*) Volcano plot showing the upregulated differentially expressed proteins (normalized to the control) according to their *p*-value versus fold change. (*b*) Proteins significantly differentially expressed (*p* ≥ 0.05) and with a fold change ≥1.

**Figure 5:**
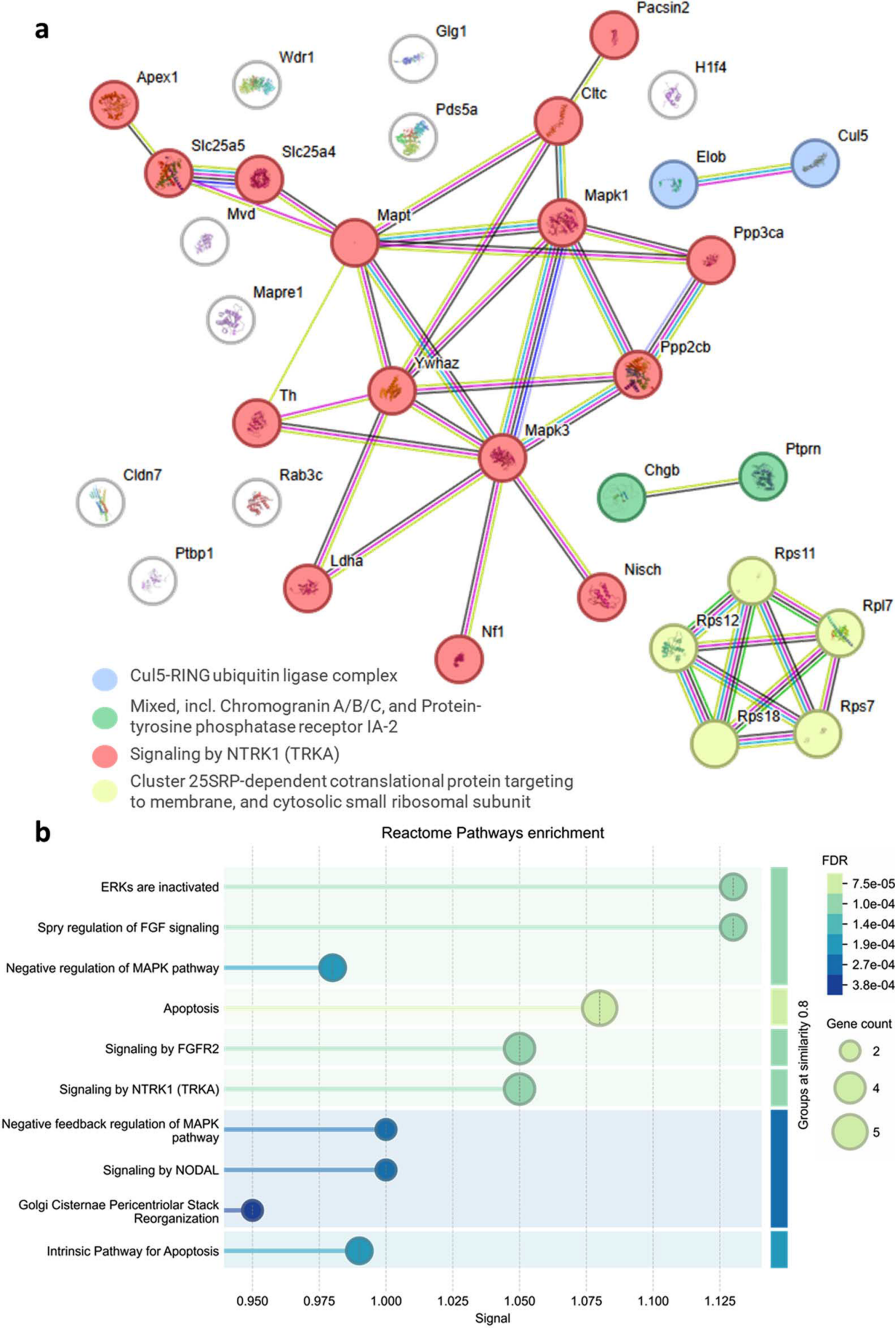
(*a*) String network analysis of differentially expressed proteins. Coloured links represent known protein–protein interactions. Cyan – from curated databases, pink – experimentally determined, green – gene neighbourhood, red – gene fusions, blue – gene co-occurrence. (*b*) Reactome pathway enrichment with pathways grouped according to similarity greater than 0.8 and sorted in terms of signal strength.

Proteomics analysis assessed the post-exposure metabolic changes to the cells in response to LI–HI THz irradiation (Fig. 4 ▸). After LI irradiation, there were only 4 proteins significantly differentially expressed compared with the control, and only one protein exhibited a log2 fold change (log2FC) of 1 or greater. By contrast, those cells exposed to MI THz irradiation showed differential expression of 33 proteins. All 33 proteins were upregulated and 31/33 proteins possessed a log2FC ≥ 1. Analysis of PC 12 cells exposed to the HI THz radiation revealed significant differential expression of 31 proteins. Of the 31 proteins identified to be differentially expressed, 16 were significantly upregulated (*p*-value < 0.05) with a log2FC ≥ 1. Additionally, upregulation of the same proteins was observed for cells exposed to all THz intensities [Fig. 4 ▸(*b*)]. Therefore, *K*-means clustering was used to identify 4 functional protein networks amongst the proteins with increased expression (Fig. 5 ▸).

The serine/threonine phosphatase calcineurin complex (CaN) PP2B (*Ppp3cb*; *Ppp3ca*) and PP2A *Ppp2cb* (log2FC > 1; *p*-value = 0.0024) were upregulated in PC 12 cells exposed to MI and HI THz.

**Figure 6:**
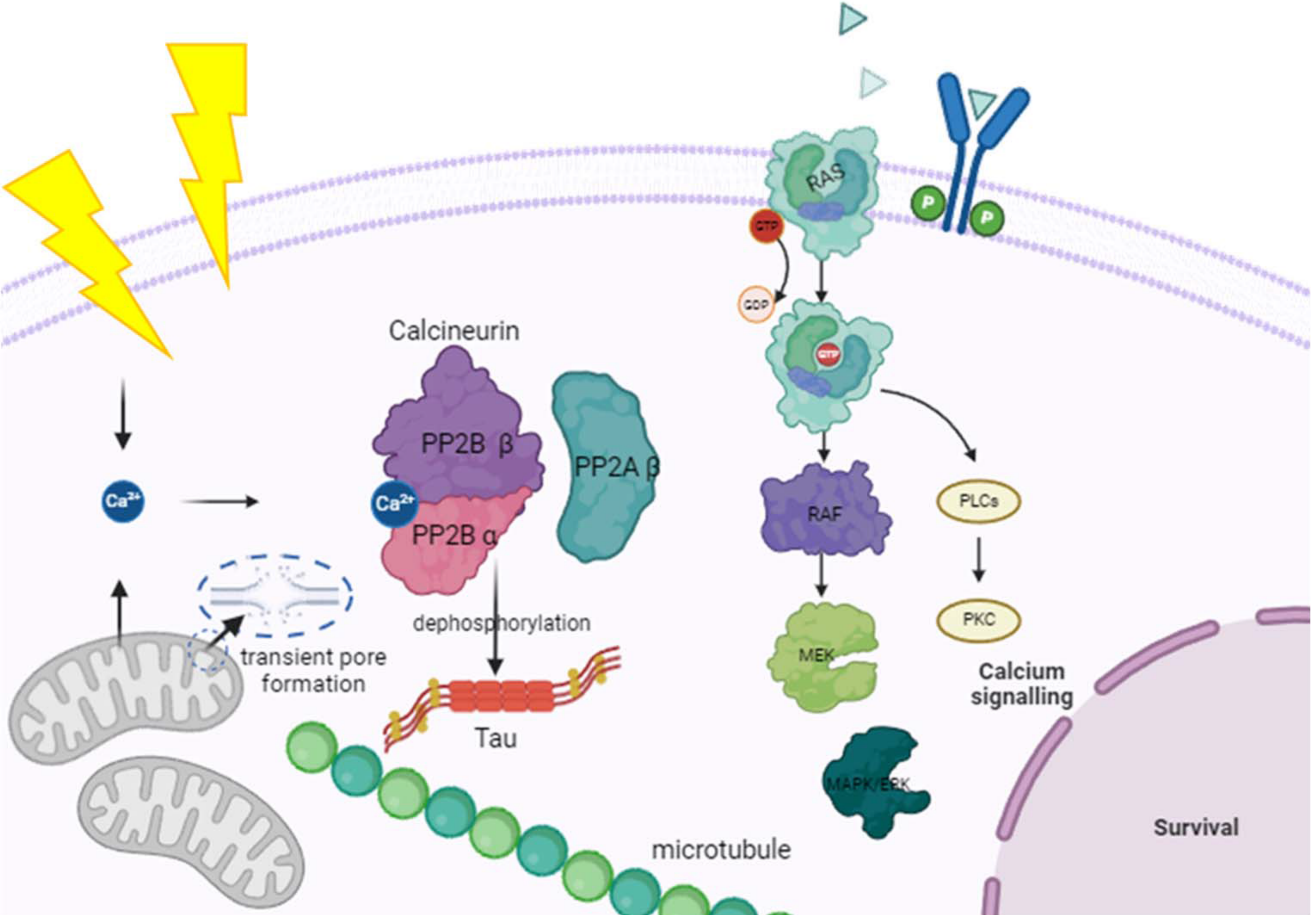
Scheme of the potential molecular/biological processes affected by non-ionizing THz radiation. The activation of the CaN complex occurs swiftly in response to an influx of intracellular calcium (Ca^2+^), playing a crucial role in the de­phospho­rylation of various important substrates. CaN is known to de­phospho­rylate the microtubule-associated proteins Mapt and MAP2. De­phospho­rylation of the Tau proteins (Mapt and MAP2) by CaN is associated with cellular response to oxidative stress (Zambrano *et al.*, 2004 ▸). Mitochondrial translocases 1 and 2 make up part of the mitochondrial permeability transition pore complex (2 out of 7 proteins). Transitional pore openings in the inner mitochondrial membrane are linked to mitochondrial dysfunction caused by mitochondrial depolarization, cessation of ATP synthesis, Ca^2+^ release, pyridine nucleotide depletion, inhibition of respiration and matrix swelling. Short-term openings may be involved in the physiological regulation of Ca^2+^ and ROS homeostasis and provide mitochondria with a fast mechanism for Ca^2+^ release, which could be a possible trigger due to SS THz radiation and as shown by previous findings where THz irradiation drove the activation of voltage-gated calcium (Ca^2+^) channels.

Within the functional networks identified in our proteomics analysis, the activation of the CaN complex may modulate the activity of the RAS/ERK/MAPK pathway (Fig. 6 ▸). Neuro­fibromin (*Nf1*), which stimulates the GTPase activity of RAS, was the most heavily upregulated protein (log2FC > 4;*p*-value = 0.0002).

Three proteins associated with the production of dense core vesicles in PC 12 neuron-like cells were heavily overexpressed in both MI and HI THz-exposed PC 12 cells including the IA-2 protein encoded by *Ptprn*, a transmembrane receptor-type tyrosine–protein phosphatase, Chromogranin B (*Chgb*), reported to be co-expressed with IA-2, and the clathrin heavy chain (Sahu *et al.*, 2017 ▸). Both *Ptprn* and *Chgb* are proteins involved in granulogenesis (formation of secretory granules) (Vethe *et al.*, 2019 ▸). They function in different aspects of cell processes – *Ptprn* has anti-apoptotic activity while *Chgb* is involved in insulin secretion (1) and clathrin is the major protein of the polyhedral coat of vesicles (Creamer, 2020 ▸) and plays a role in early autophagosome formation.

Fifteen proteins related to signalling by NTRK1 (Trk tropomyosin receptor kinase) were upregulated in PC 12 exposed to THz radiation (Fig. 5 ▸). STRING analysis resulted in a hypothetical functional network connecting NTRK1 via activation of the RAS/MAPK/ERK pathway and protein kinase C. Activation of RAS mediates neurotrophin-induced survival and differentiation. Of the 15, there were four up­regulated proteins identified to belong to the stress-activated MAPK cascade including *Nf1*, *Mapk1*, *Mapk3* and *Ywhaz*. Activation of the RAS/MAPK/ERK pathway also triggers a signal link to protein kinase receptors (Creamer, 2020 ▸). Herein, protein kinase C *pacsin 2* was additionally upregulated. Other proteins not functionally linked to the network include RAS-related protein Rab3C which plays a role in vesicular traffic (Ma *et al.*, 2022 ▸). We also observed the upregulation of the Histone H1 protein. Sister chromatid cohesion protein *Pds5a* was similarly upregulated which plays an important role in DNA repair. One protein making up the Cullin-RING ligase 5 *Cul5* was overexpressed. The final functional pathway to be upregulated were genes encoding ribosomal proteins for the 40S and 60S ribosomal subunits. The upregulation of genes involved in ribosome biogenesis has previously been reported for neurons exposed to THz irradiation (Shang *et al.*, 2021 ▸).

## 4. Discussion

In this study, SS THz radiation was delivered as a continuous wave with an incident power density of 0.25–1 W m^−2^. A significantly increased uptake of FITC–dextran and FITC–SiO_2_ NS was observed in those cells exposed to HI (1 W m^−2^) THz. In a similar study, at a THz frequency of 2.3 (0.5–20 mW cm^−2^), neurons experienced a dose-dependent increase in membrane permeability. The authors proposed that THz radiation induced the formation of transient hydro­philic pores in the membrane. In prior work, localization of the NS was performed, establishing that the majority of the NS are present in the cytoplasm and are sequestered in vacuoles (Perera *et al.*, 2023 ▸; Perera, Bazaka *et al.*, 2019 ▸; Perera *et al.*, 2018 ▸). These results corroborate our current observation of reversible permeation of the membrane of PC 12 cells, leading to the uptake of NS (20–40 nm diameter) and FITC–dextran. Moreover, similar studies have posited that the formation of hydro­philic pores in the cell membrane may be induced by THz-induced oxidative stress, as antioxidants could prevent this process (Zapara *et al.*, 2015 ▸).

Indeed, multiple reports have demonstrated that THz irradiation does not induce the enhanced generation of reactive oxygen species (ROS) (Sitnikov *et al.*, 2021 ▸), *e.g.* irradiation of human fibroblasts with super HI THz (GW cm^−2^) for extended periods did not lead to the production of exogenous ROS or heat shock. Rather, recent research efforts (summarized in Table 2 ▸) have illuminated that THz EMF irradiation can activate voltage-gated calcium (Ca^2+^) channels, voltage-gated potassium (K^+^) channels and active transport Ca^2+^ channels in the cell membrane and create hydro­philic pores in the phospho­lipid membrane of the cell membrane, leading to enhanced cell permeability (Romanenko *et al.*, 2020 ▸; Romanenko *et al.*, 2014 ▸; Shang *et al.*, 2021 ▸). Surprisingly, we observed perturbation of the cell membrane of cells exposed to LI THz through the formation of membrane blebs. Cell blebbing (zeiosis) is a bulge of the plasma membrane due to high intracellular pressure and disruption of the membrane-actin cortex interactions (Price, 1967 ▸). In some cases, blebbing is an early phenotypic indication of apoptosis (Deschesnes *et al.*, 2001 ▸). We previously hypothesized that the cell blebbing in response to THz EMF may be a cell response to maintain cell membrane stability through increased volumes of cholesterol and regulation of the synthesis of actin/actin-related proteins (Perera, Bazaka *et al.*, 2019 ▸; Perera *et al.*, 2022 ▸; Perera *et al.*, 2018 ▸; Perera *et al.*, 2023 ▸). At higher THz intensities, SEM and CLSM imaging revealed further changes in cell morphology such as reduced cellular area potentially caused by the loss of cytosolic material.

In the current study, we propose that THz irradiation may lead to an increase in intracellular Ca^2+^ as proteomics analysis revealed the activation of the CaN complex (Fig. 6 ▸) (Bo *et al.*, 2021 ▸). An increase in intracellular Ca^2+^ in neurons exposed to 0.1 THz with a power density of 2.65 mW cm^−2^ was previously reported (Zhang *et al.*, 2020 ▸). In addition to Ca^2+^, Na^+^ concentrations increased, and the K^+^ concentration decreased after irradiation by the THz source. In experiments of 3D human skin tissue models exposed to intense THz pulses, there was major down-regulation of expression of genes involved in tubulin formation [especially principal components of microtubules (MTs)]; however, MAP family genes were upregulated. The authors predicted that differential gene expression may represent the cell’s genomic response to a disassembled and disrupted cytoskeletal network (Hough *et al.*, 2021 ▸). *In vitro* experiments revealed the disassembly of MTs within 11 min of exposure to intense (18 ± 3 mW cm^−2^), picosecond-duration 0.5–1.5 THz pulses. Further, the dis­assembly rate depended on THz intensity and spectral content (Hough *et al.*, 2021 ▸). In this study, the overexpression of Tau protein (isoform unknown) and the significantly increased activity of the CaN complex may be an effort to de­phospho­rylate the MT-associated protein to stabilize the cell following a stress assault as Tau protein in its de­phospho­rylated form binds more efficiently to MTs and cell membranes (Zambrano *et al.*, 2004 ▸; Sommer *et al.*, 2002 ▸). Thus, their overabundance following THz irradiation may be due to prior inhibition and then recovery, or in response to hyperphospho­rylation.

Our recent analysis of the FTIR spectra of THz exposed cells revealed variations in the amide I and amide II bands (Perera *et al.*, 2025 ▸). The variation in the secondary structure of protein bands may reflect changes in actin-related proteins. Other changes detected by FTIR in PC 12 cells exposed to SS THz were related to changes in lipid composition and the stretching vibration of phosphate groups found in the phospho­diester backbone of DNA and RNA.

Many studies of the cell response to THz irradiation report DNA changes (Wang *et al.*, 2023 ▸; Zhao *et al.*, 2023 ▸; Shang *et al.*, 2021 ▸; Sitnikov *et al.*, 2021 ▸; Bock *et al.*, 2010 ▸). For example, HI THz (GW cm^−2^) radiation caused phospho­rylation of the histone in human fibroblast cells but did not affect their proliferative activity or morphology (Sitnikov *et al.*, 2021 ▸). Histone (H2A.X) staining of foci was used to confirm DNA double-stranded breaks (Wang *et al.*, 2023 ▸; Titova *et al.*, 2013 ▸). By contrast, other studies report no changes to DNA and no upregulation of genes involved in DNA damage repair in response to 0.3–2.5 THz radiation (Hintzsche *et al.*, 2013 ▸; Wilmink *et al.*, 2011 ▸; Alexandrov *et al.*, 2013 ▸; Bogomazova *et al.*, 2015 ▸). When DNA damage occurs, the DNA damage response (DDR) involves the signalling of a cascade of protein kinases and the activation of a series of downstream effectors that promote cell cycle arrest, DNA repair or activation of apoptotic pathways (Nagelkerke & Span, 2016 ▸). Here, the RAS/MAPK/ERK pathway and stress-activated MAPK cascade were upregulated in THz-exposed PC 12 cells. The MEK/ERK pathway is activated during DDR, which contributes to the appropriate activation of checkpoints to inhibit cell division (Toral, 2021 ▸; Nestler & Greengard, 1999 ▸). Additionally, our proteomics analysis revealed the overexpression of histone, protein kinase C and casein kinase, which may point to the event of DDR in THz-irradiated cells. CLSM and FC results further established that DNA changes were incurred because of HI THz exposure in PC 12 cells. However, there was no significant immediate loss of cell viability; thus, the cells were believed to promote DNA repair and cell survival, rather than apoptosis. In agreement with our study, HI pulsed THz irradiation was shown to promote DNA damage in human skin tissue; however, the upregulation of multiple tumour suppressor proteins was suggested to be indicative of the THz-pulse-induced DNA damage being effectively repaired (Titova *et al.*, 2013 ▸). While THz photons lack the energy required to directly influence chemical reactions, nonlinear resonance effects can induce localized changes in the breathing dynamics of DNA, potentially affecting gene transcription. The hydrogen bonds in double-stranded DNA (dsDNA) vibrate at THz frequencies, which means THz radiation could potentially impact crucial cellular functions related to genomic DNA and DNA–protein interactions (Bock *et al.*, 2010 ▸).

Similar work reporting the gene expression changes of neurons exposed to 0.1 THz irradiation for 20 min with an average power density of 33 mW cm^−2^ showed significant enrichment in the categories of biomacromolecule interaction processes such as GTPase binding, phospho­lipid binding, tropomyosin binding, BMP receptor binding and long-chain fatty acid binding. The authors concluded that THz irradiation possibly affected cell mitosis, protein transcription and translation, phospho­lipid distribution, and the biomacro­molecule interaction processes. In corroboration with the current study, KEGG signalling pathway analysis also indicated that the differentially expressed genes mainly participated in the calcium signalling pathway (Shang *et al.*, 2021 ▸). Additionally, another study identified upregulated gene expression for the regulation of cell mitosis, cellular response to calcium ions, MT assembly and neuronal projections (Zhao *et al.*, 2021 ▸) when neurons were exposed to 0.07 mW cm^−2^ 3.1 THz for 3 h.

Furthermore, studies of the effects of continuous-wave laser THz radiation of 60 min duration at broadband frequencies (0.71–4.28 THz; intensity 2–20 mW cm^−2^) on isolated ganglial cells revealed changed adhesive properties of cell membranes and disrupted contacts between the neurons and the support. Longer-wave THz radiation caused damage to neurite growth cones and arrested their further growth 40–50 h after irradiation (Olshevskaya *et al.*, 2008 ▸), limiting neurite growth. In our previous study, 86.2 ± 4.0% of THz-treated PC 12 cells underwent neuronal differentiation compared with 65.9 ± 5.0% of the untreated control cells (Perera, Bazaka *et al.*, 2019 ▸). After 7 days post-exposure to SS THz, differentiated PC 12 cells had a reduced number of neurite outgrowths and length (Perera, Bazaka *et al.*, 2019 ▸; Zhao *et al.*, 2023 ▸). We previously observed increased metabolic activity (evidence of increased mitochondrial function) in PC 12 cells exposed to THz radiation in comparison with the untreated control (Perera, Bazaka *et al.*, 2019 ▸). We assume our prior results are attributed to an acute stress response, leading to upregulating calcium ions and enhancing enzyme activity. Research on THz-exposed neural progenitor cells (drNPCs) and neuroblastoma cells (SK-N-BE) did not reveal any differences in proliferative activity or the occurrence of double-strand DNA breaks (Shatalova *et al.*, 2021 ▸).

## 5. Conclusions

In summary, THz radiation of different power densities causes disparate and complex responses in PC 12 cells. For example, 10 min exposure to THz of 0.25 W m^−2^ induced the formation of membrane blebbing in the neuron-like cells; however, the cells were not apoptotic and remained viable. At higher incident power densities (1 W m^−2^), the cells were shown to be transiently permeable to dextran (17 nm) and SiO_2_ NS (25 nm). DNA changes were observed at 1 W cm^−2^. Proteomics analysis indicated that, after exposure, the cells initiated DNA reparation rather than triggering the cell apoptosis cascade.

## Supplementary Material

Supporting figures and table. DOI: 10.1107/S1600577525008227/kam5008sup1.pdf

## Figures and Tables

**Table 1:** Beam intensity settings

	Mirror position at BEP (mm)	Si Bolometer count (arb. units)	Beam intensity at the sample (W m^−2^)
High (HI)	9.5	500	1.00
Medium (MI)	6.5	11400	0.50
Low (LI)	5.5	17000	0.25

**Table 2:** Exposure of cell components and neuronal cells to continuous-wave THz radiation

	Observation	Time exposure	Incident power density (mW cm^−2^)	THz frequency	Reference
Biomolecules					
Tryptophan (Trp) amino acid	Fluorescence signal quenched by ∼54%	90 s	11700	2.55	Kaur & Zhang (2014 ▸)
Whey proteins	Drop of the fluorescence signal by ∼10%	90 s	140	0.2	Kaur & Zhang (2014 ▸)
Green fluorescent protein	Fluorescence enhancement by ∼5%	90 s	120	0.2	
Lysozyme	Electron density local increase in a long α-helix motif	25 ms	62	0.4	Lundholm *et al.* (2015 ▸)
Bovine serum albumin	Modifications in the BSA conformation	60 min	20	3.67	Cherkasova *et al.* (2009 ▸)
Alkaline phosphatase	Reduction in enzyme activity	90–120 min	0.008	0.1	Homenko *et al.* (2009 ▸)

Neuronal cell					
PC 12 pheochromocytoma cells	Cell blebbing at 0.025 mW cm^−2^	10 min	0.025–0.1	0.5–20	This study.
	Cell permeabilization at 0.1 mW cm^−2^				
	DNA structural changes at 0.1 mW cm^−2^				
*L. stagnalis* neurons	Alteration of adhesive properties of neuron membrane	60 min	10–20	0.71	Olshevskaya *et al.* (2008 ▸)
	Alteration of neuron membrane and structural changes of the somatic membrane, axons and growth cone	60 min	10–20	3.68	
Glial cells	Number of apoptotic cells increased 2.4-fold	5 min	3.2	0.12–0.18	Borovkova *et al.* (2016 ▸)
Retzius neurons of leech	Dose-dependent increase in the plasma membrane permeability	1 min	0.1–0.6	0.06	Pikov & Siegel (2011 ▸)
Primary hippocampal neurons	Changes to the synapse function and calcium signalling pathway	20 min	33	0.1	Shang *et al.* (2021 ▸)
The leech ganglia neuron	Upregulation of free intracellular calcium	1 min	100	0.06	Romanenko *et al.* (2014 ▸)
Primary hippocampal neuronal cells	THz induced apoptosis and altered cellular activity	10–30 min	10–50	0.12–0.15	Romanenko *et al.* (2020 ▸)
	Release of amino acid neurotransmitter that affected the homeostasis of neuronal excitability				
Neural stem cells	Inhibition of proliferation and increased DNA damage and apoptosis of neural stem cells as a function of THz output power and irradiation time	5–10 min	25–50	0.22	Wang *et al.* (2023 ▸)
Cortical primary neurons	THz promoted the growth of neuronal cytosomes and protrusions	3 min	0.1	0.1–2	Shaoqing *et al.* (2023 ▸)
Primary hippocampal neurons	THz radiation altered the binding activity of AP-1 with its DNA probe	20 min	33	0.1	Shang *et al.* (2021 ▸)
	Significant enrichment of genes involved in GTPase binding, phospho­lipid binding, tropomyosin binding, BMP receptor binding and long-chain fatty acid binding				
	Significant upregulation of genes involved in ribosome biogenesis and the calcium signalling pathway				
Mouse primary cortical neurons and oligodendrocytes	THz increased excitatory synaptic activities of neurons	3 h	0.07	3.1	Zhao *et al.* (2021 ▸)
	Enhanced neurite outgrowth				
	Upregulated genes associated with ‘neuron projection’, ‘synapse organization’ and ‘dendritic spine’				

## Data Availability

The data supporting the findings of this study are available within the paper and its supporting information. Should any raw data files be needed in another format they are available from the corresponding author upon reasonable request.
