# Supplementary material for: Response of pheochromocytoma neuronal cells to varying intensity of continuous wave terahertz radiation
Source: J Synchrotron Radiat. 2025 Oct 16;32(Pt 6):1431–44. doi: 10.1107/S1600577525008227 (PMC12591077; doi:10.1107/S1600577525008227)
Supplement: Supplementary file 1 [file s-32-01431-sup1.pdf]

JOURNAL OF SYNCHROTRON RADIATION

Volume 32 (2025)

Supporting information for article:

Response of Pheochromocytoma Neuronal Cells to Varying Intensity of Continuous Wave Terahertz Radiation

Denver P. Linklater, Palalle G. Tharushi Perera, Zoltan Vilagosh, Alexis Perez-Gonzales, Phuc H. Le, Tanavi Sharma, Michael G. Leeming, Nicholas A. Williamson, Dominique Appadoo, Rodney Croft and Elena P. Ivanova

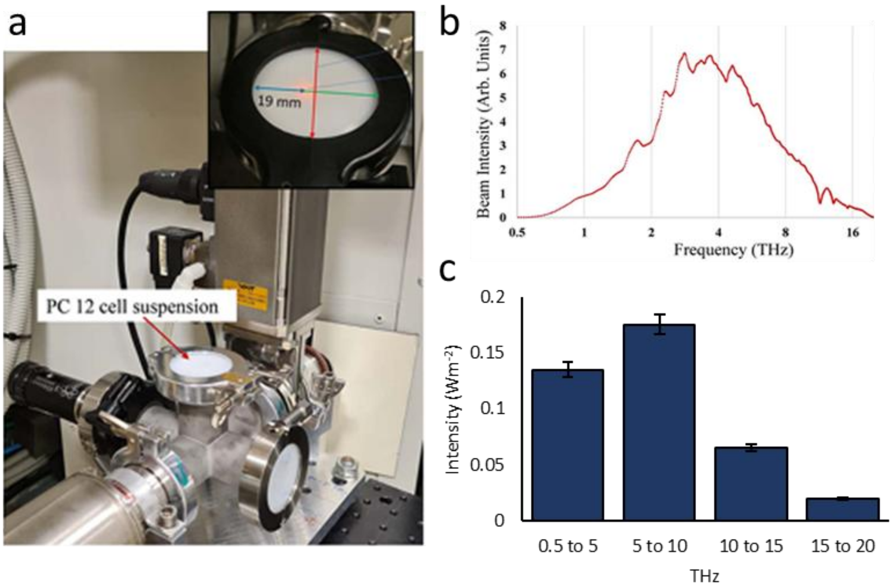

Figure S1 (a) Photograph of the experimental setup at the Australian Synchrotron Far-IR/ THz beamline. The inset shows the beam axis in red; the beam is present within a 19 mm diameter (blue arrow) from the inner edge to the beam axis. The PC 12 cell suspension was placed on a polyethylene window (red arrow) directly over the beam. (b) The distribution of the intensity of the THz beam as shown by the resident Si bolometer. (c) Absorption pattern of the THz beam according to THz frequency, assuming a sample depth of 5-10 µm. The absorption of the beam decreases as the depth of the sample increases.

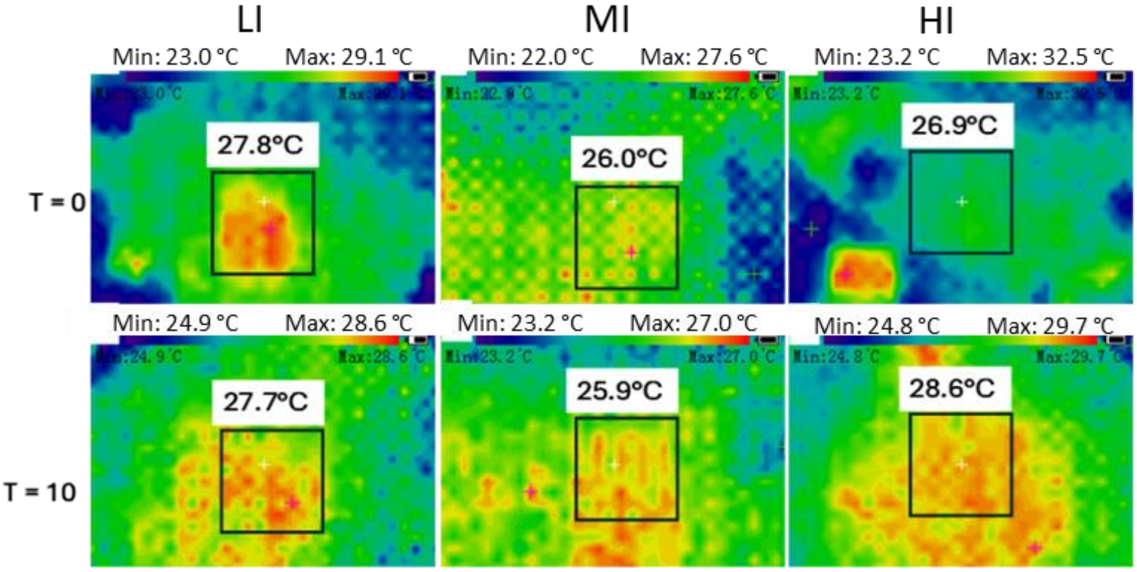

Figure S2 IR heat maps illustrating the recorded temperatures at T=0 min and T=10 min at low, medium, and high intensities, respectively. The black boxes indicate the position of the cell suspension in the image. There were no statistically significant differences in the recorded temperature before (at T = 0 min) and following (at T = 10 min).

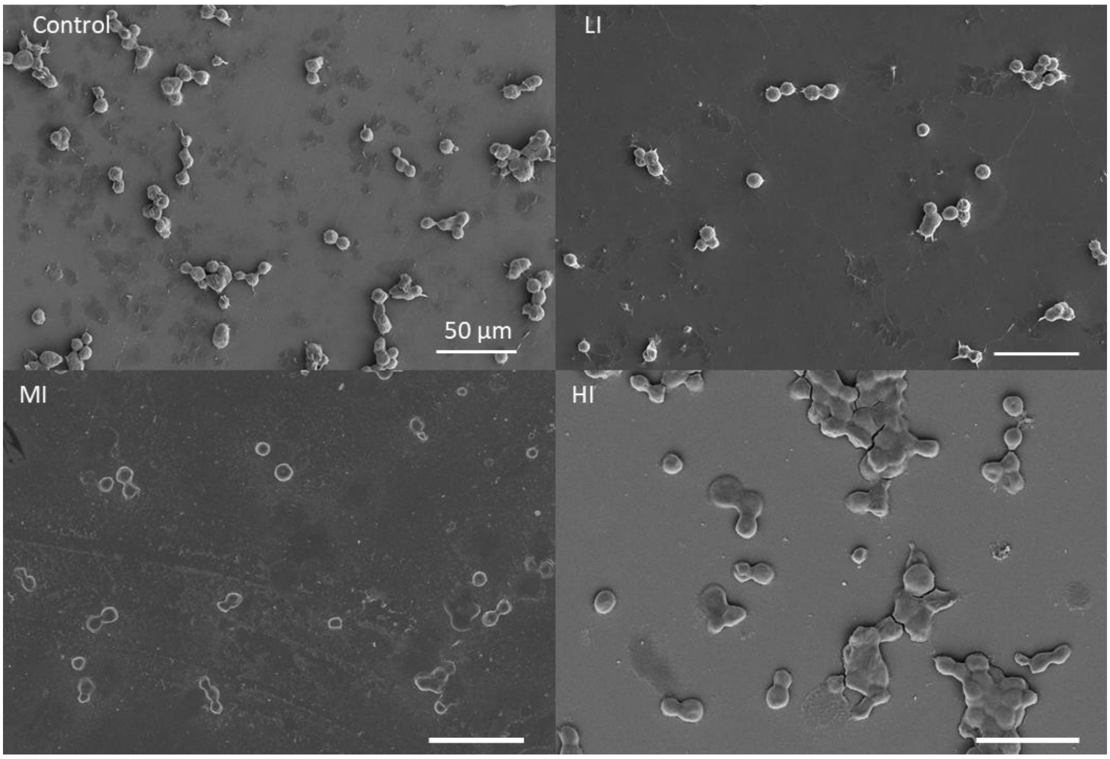

Figure S3 Low magnification SEM micrographs of PC 12 cells following exposure to LI, MI, and HI THz for 10 min.

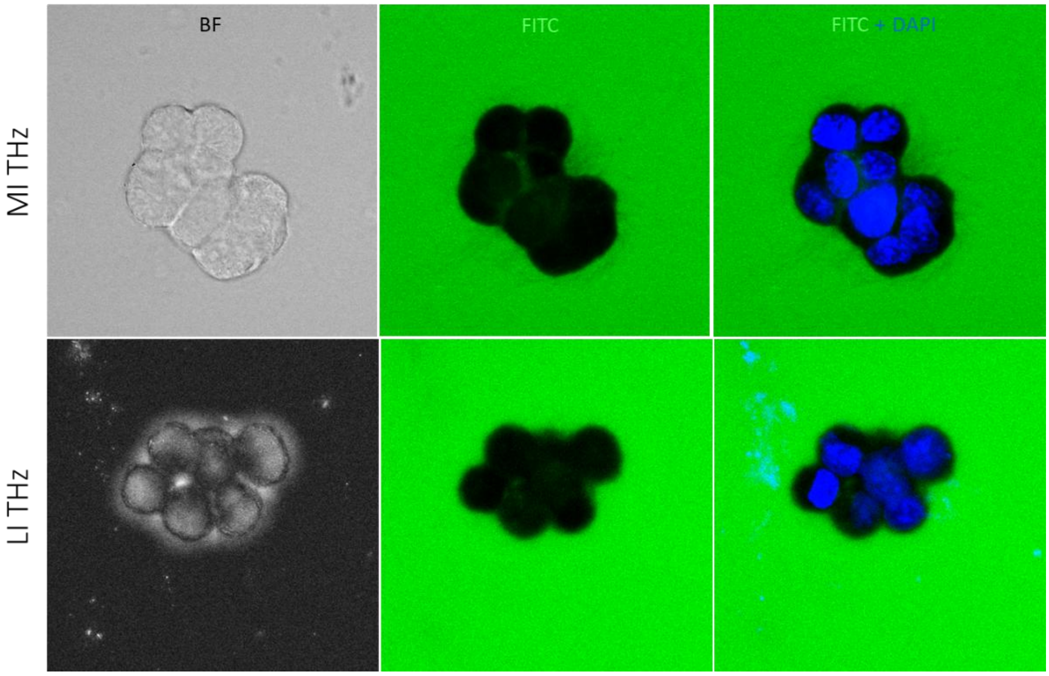

Figure S4 Fluorescein dextran uptake by PC 12 cells exposed to low, mid-intensity THz for 10 min. CLSM micrographs of cells treated with LI and MI intensity THz and immediately placed in fluorescein dextran.

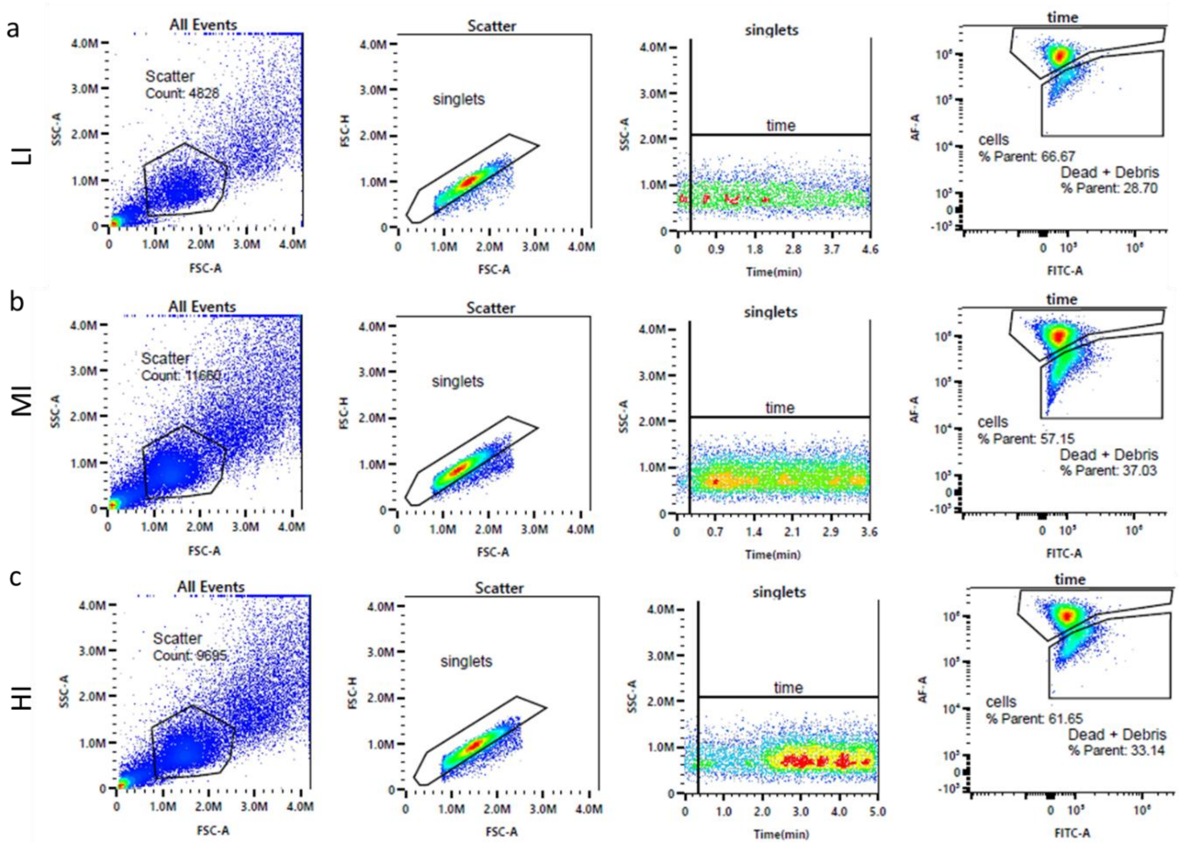

Figure S5 Flow cytometry scatter plots showing the population of single live cells exposed to LI, MI and HI THz.

Table S1 Differentially expressed proteins and their protein IDs and corresponding gene name.

Majority protein IDs	Protein names	Gene names
A4L9P7	Sister chromatid cohesion protein PDS5 homolog A	Pds5a
O35314	Secretogranin-1;PE-11;CCB peptide short form;CCB peptide long form	Chgb
P04177	Tyrosine 3-monooxygenase	Th
P04642	L-lactate dehydrogenase A chain	Ldha
P05426	60S ribosomal protein L7	Rpl7
P11442	Clathrin heavy chain 1	Cltc
P15865	Histone H1.4	Hist1h1e
P19332	Microtubule-associated protein tau	Mapt
P21708	Mitogen-activated protein kinase 3	Mapk3
P43138	DNA-(apurinic or apyrimidinic site) lyase;DNA-(apurinic or apyrimidinic site) lyase, mitochondrial	Apex1
P62083	40S ribosomal protein S7	Rps7
P62271	40S ribosomal protein S18	Rps18
P62282	40S ribosomal protein S11	Rps11
P62716	Serine/threonine-protein phosphatase 2A catalytic subunit beta isoform	Ppp2cb
P62824	Ras-related protein Rab-3C	Rab3c
P63086	Mitogen-activated protein kinase 1	Mapk1
P63324	40S ribosomal protein S12	Rps12
P63329	Serine/threonine-protein phosphatase 2B catalytic subunit alpha isoform	Ppp3ca
P97526	Neurofibromin	Nf1
Q00438	Polypyrimidine tract-binding protein 1	Ptbp1
Q05962	ADP/ATP translocase 1	Slc25a4
Q09073	ADP/ATP translocase 2;ADP/ATP translocase 2, N-terminally processed	Slc25a5
Q4G017	Nischarin	Nisch
Q5RKI0	WD repeat-containing protein 1	Wdr1
Q62638	Golgi apparatus protein 1	Glg1
Q62967	Diphosphomevalonate decarboxylase	Mvd

Q63259	Receptor-type tyrosine-protein phosphatase-like N	Ptprn
Q9JJ31	Cullin-5	Cul5
Q9QY17	Protein kinase C and casein kinase substrate in neurons 2 protein	Pacsin2
Q9Z1L1	Claudin-7	Cldn7
P62870	Transcription elongation factor B polypeptide 2	Tceb2
P63102	14-3-3 protein zeta/delta	Ywhaz
Q66HR2	Microtubule-associated protein RP/EB family member 1	Mapre1
